# Supplementary material for: Inhibiting Infectious Bronchitis Virus PLpro Using Ubiquitin Variants
Source: Int J Mol Sci. 2025 May 29;26(11):5254. doi: 10.3390/ijms26115254 (PMC12155308; doi:10.3390/ijms26115254)
Supplement: Supplementary file 1 [file ijms-26-05254-s001.zip › ijms-3622843-supplementary.pdf]

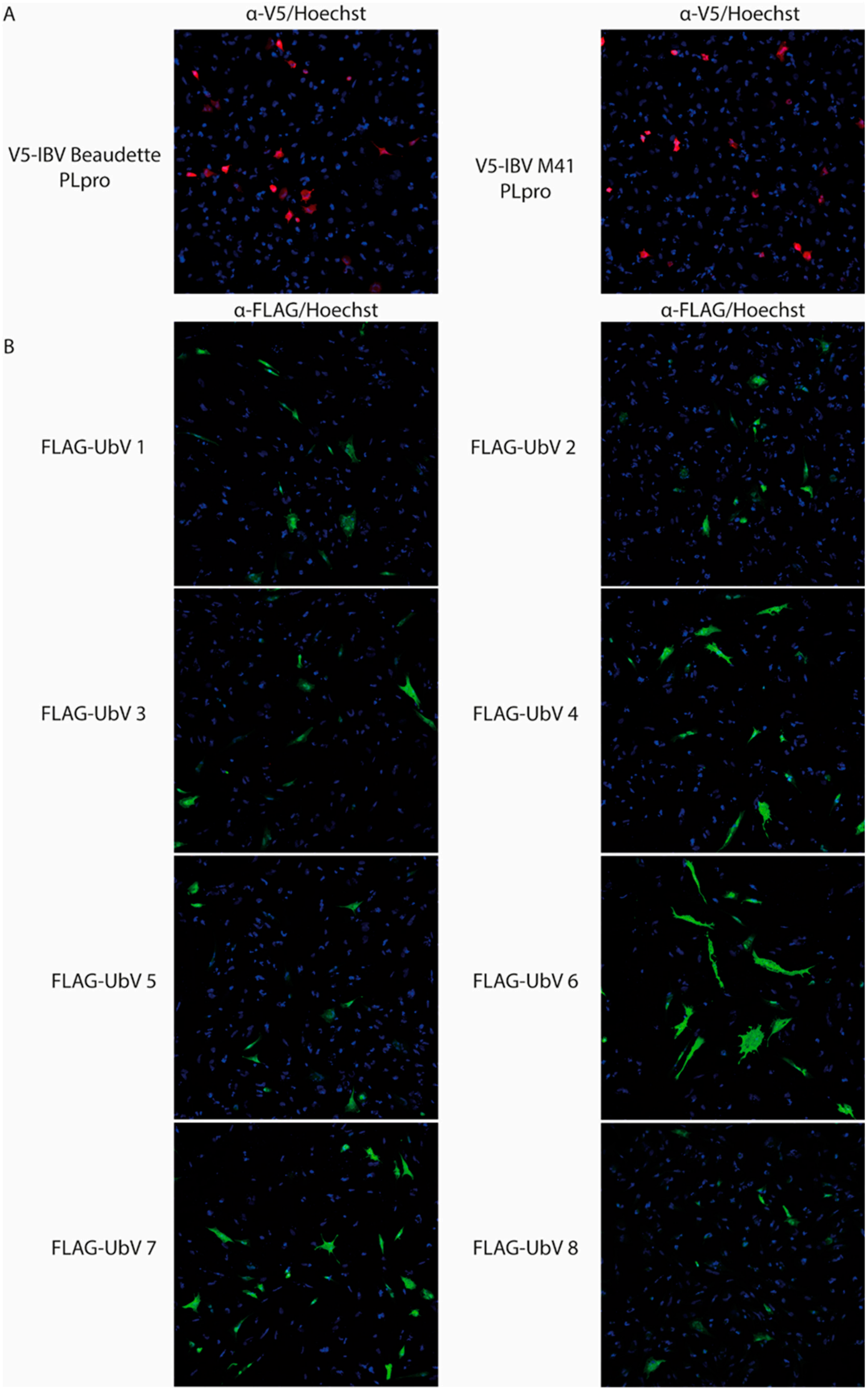

Figure S1. Expression of IBV PLpro and UbVs. Expression of IBV Beaudette (Beau) and M41 PLpro as detected using immunofluorescence assay (**A**). PLpro is shown in red (V5), nuclei in blue (Hoechst). (**B**) Expression of UbVs 1-8, detected in the same manner, with FLAG (UbV) expression in green and nuclei in blue (Hoechst), 20× magnification.

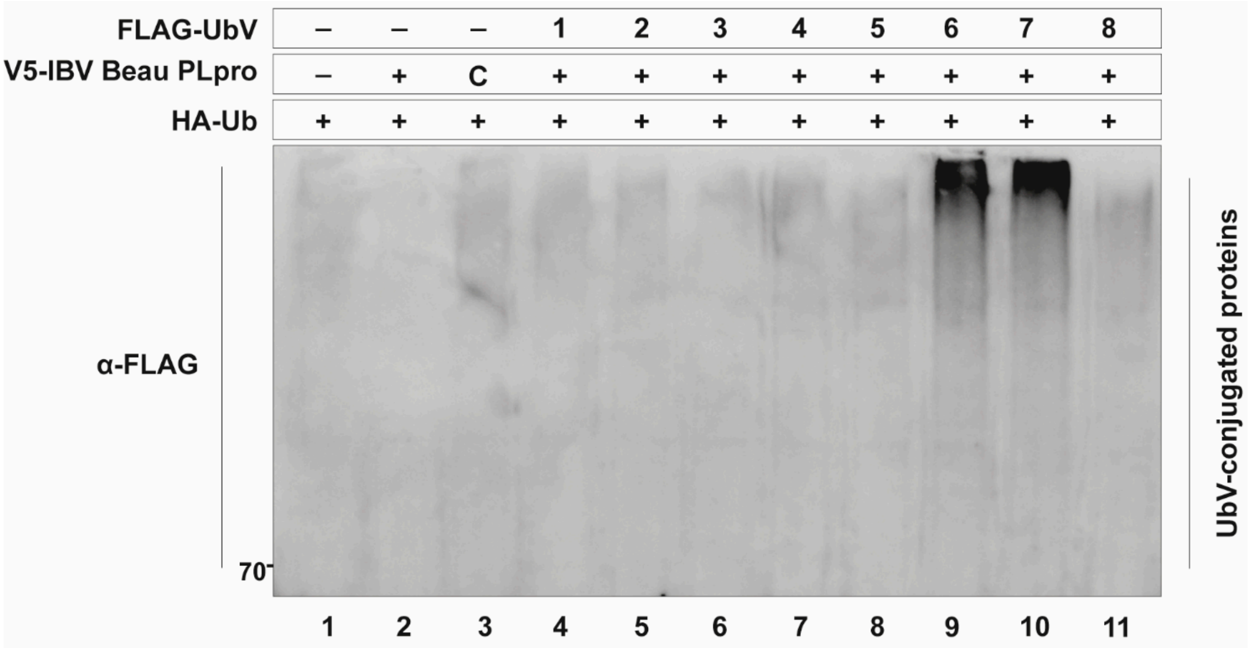

Figure S2. UbVs 6 and 7 are conjugatable to cellular proteins. Transfection of DF-1 cells with plasmids expressing the different UbVs show an ability of UbVs 6 and 7 to be conjugated to different cellular substrates, causing a visible smear of conjugated UbV on western blot.

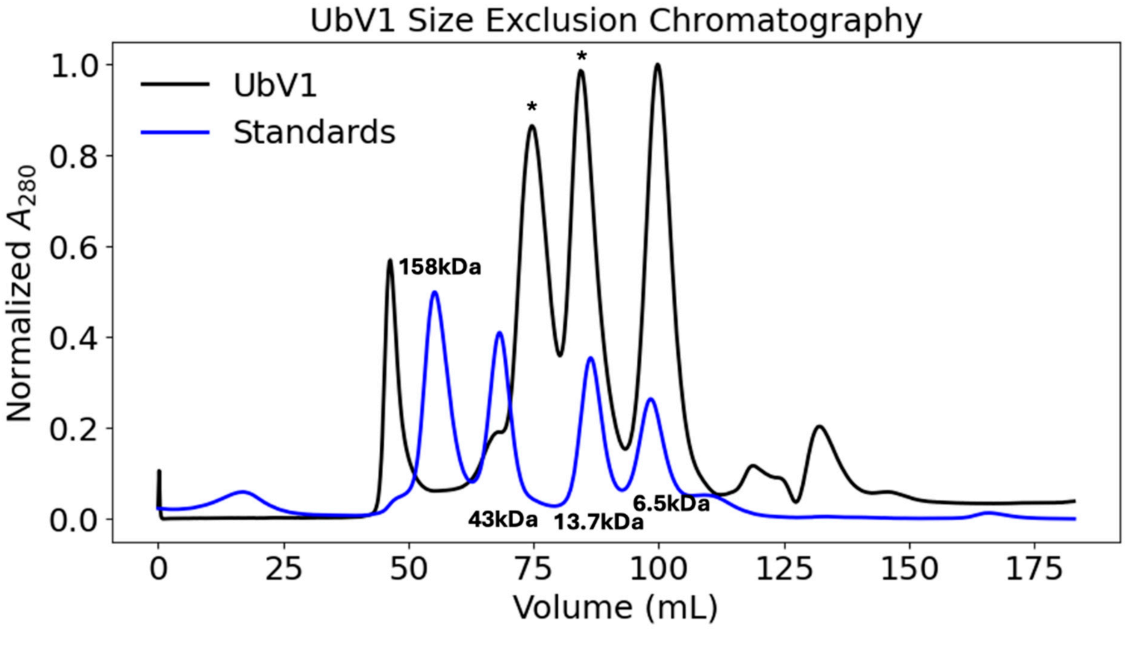

Figure S3. Size exclusion chromatography profile of UbV 1. According to the calibration profile of standards, two peaks labelled with asterisks correspond to dimer and monomer sizes, respectively. The first peak is shows aggregates, while the fourth peak appears to be degraded UbV. The fractions corresponding to the first and fourth peaks were excluded based on their size as assessed by SDS-PAGE.

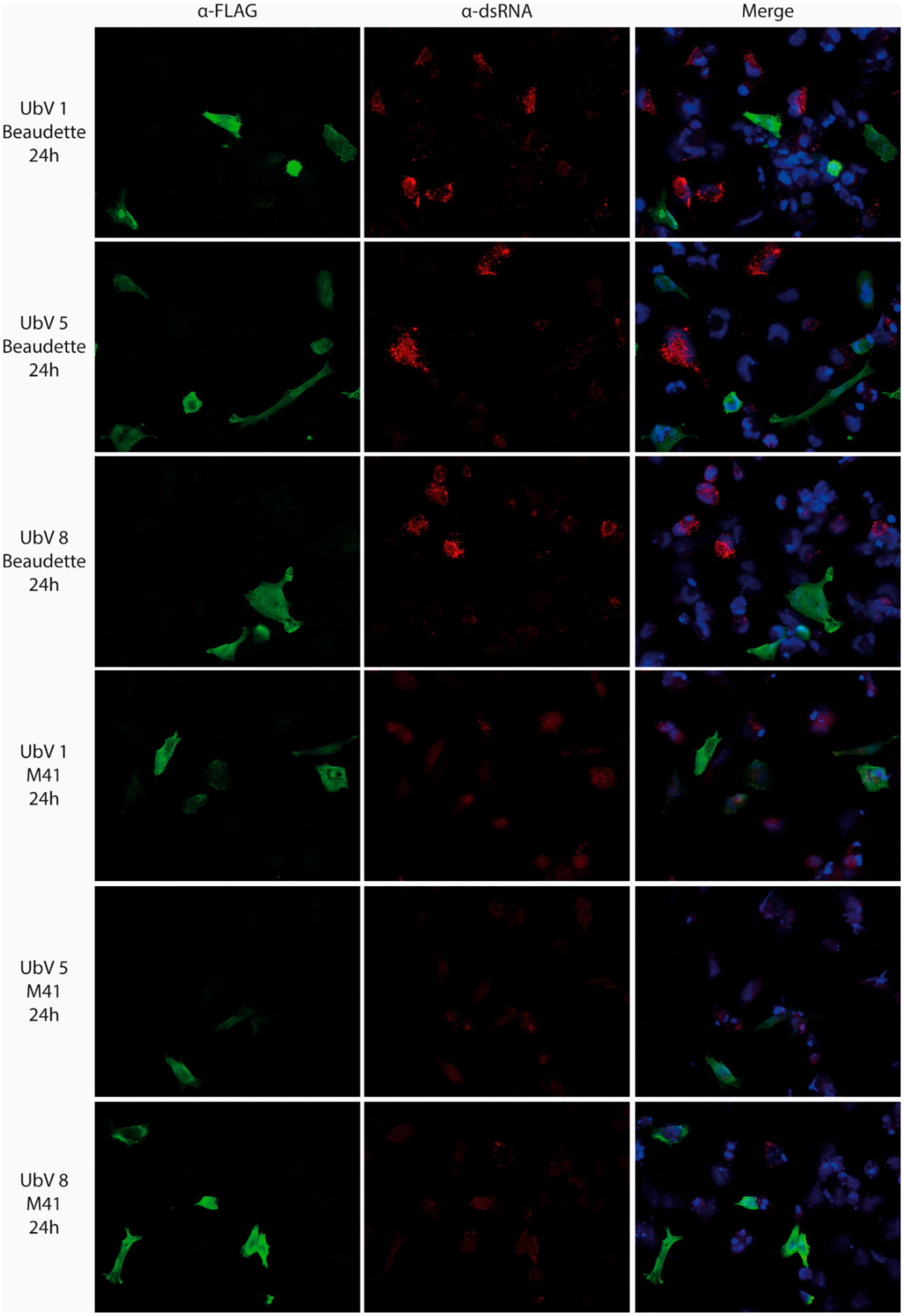

Figure S4. Remaining UbVs inhibit IBV infection. Immunofluorescence was performed to examine double stranded RNA (dsRNA) expression and thus viral infection in control (GFP) and UbV 1, 5 or 8-expressing DF-1 cells infected with IBV Beaudette or IBV M41 and fixed at 24 hpi. Viral staining (dsRNA) is shown in red, GFP and FLAG-UbV is shown in green, and Hoechst depicted in blue, 40× magnification.

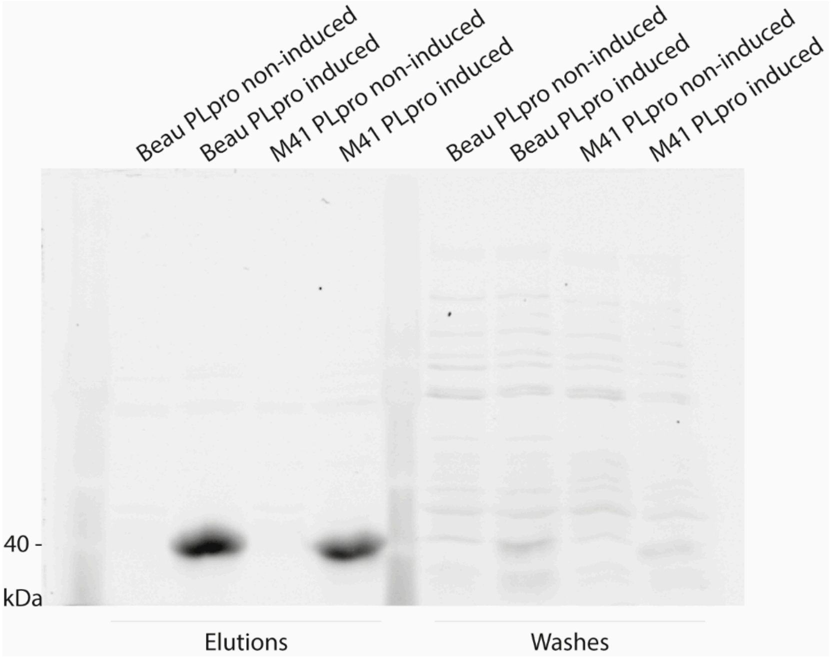

Figure S5. Expression and purification of IBV Beaudette and M41 PLpro. Elutions on the left show expressed and purified Beaudette and M41 PLpro, at their expected size. Washes on the right show background proteins washed off before elution.
